# Supplementary material for: Using high-resolution contact networks to evaluate SARS-CoV-2 transmission and control in large-scale multi-day events
Source: Nat Commun. 2022 Apr 12;13:1956. doi: 10.1038/s41467-022-29522-y (PMC9005731; doi:10.1038/s41467-022-29522-y)
Supplement: Supplementary file 1 — Supplementary Information [file 41467_2022_29522_MOESM1_ESM.pdf]

Supplementary Information

Using high-resolution contact networks to evaluate SARS-CoV-2 transmission and control in large-scale multi-day events

Rachael Pung*, Josh A Firth, Lewis G Spurgin, Singapore CruiseSafe working group, CMMID COVID-19 working group, Vernon J Lee, Adam J Kucharski

*Correspondence to: rachael.pung@lshtm.ac.uk

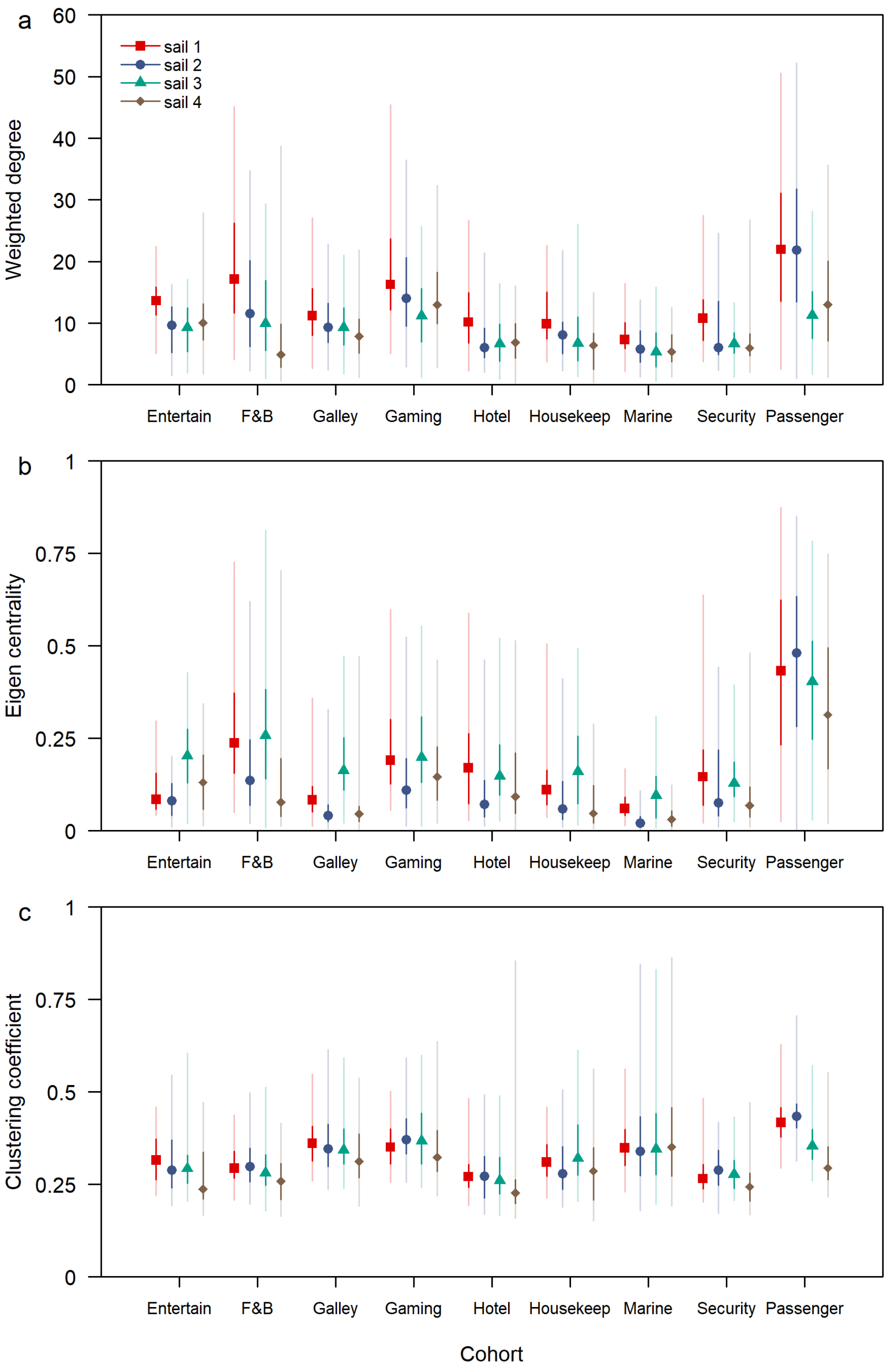

Supplementary Figure 1 Social network analysis over four cruise sailings. (a) Weighted degree, (b) eigenvector centrality, (c) clustering coefficient of crew and passengers of each sailing. Colours represent the cruise departure date and the median (shapes), 50% (dark lines) and 95% intervals (light lines) network property measures from 5,216 passenger and 4,197 crew across 4 sailings are shown. Weights were assigned based on exponent transformation of the mean daily cumulative duration of interaction between two individuals (see Methods)

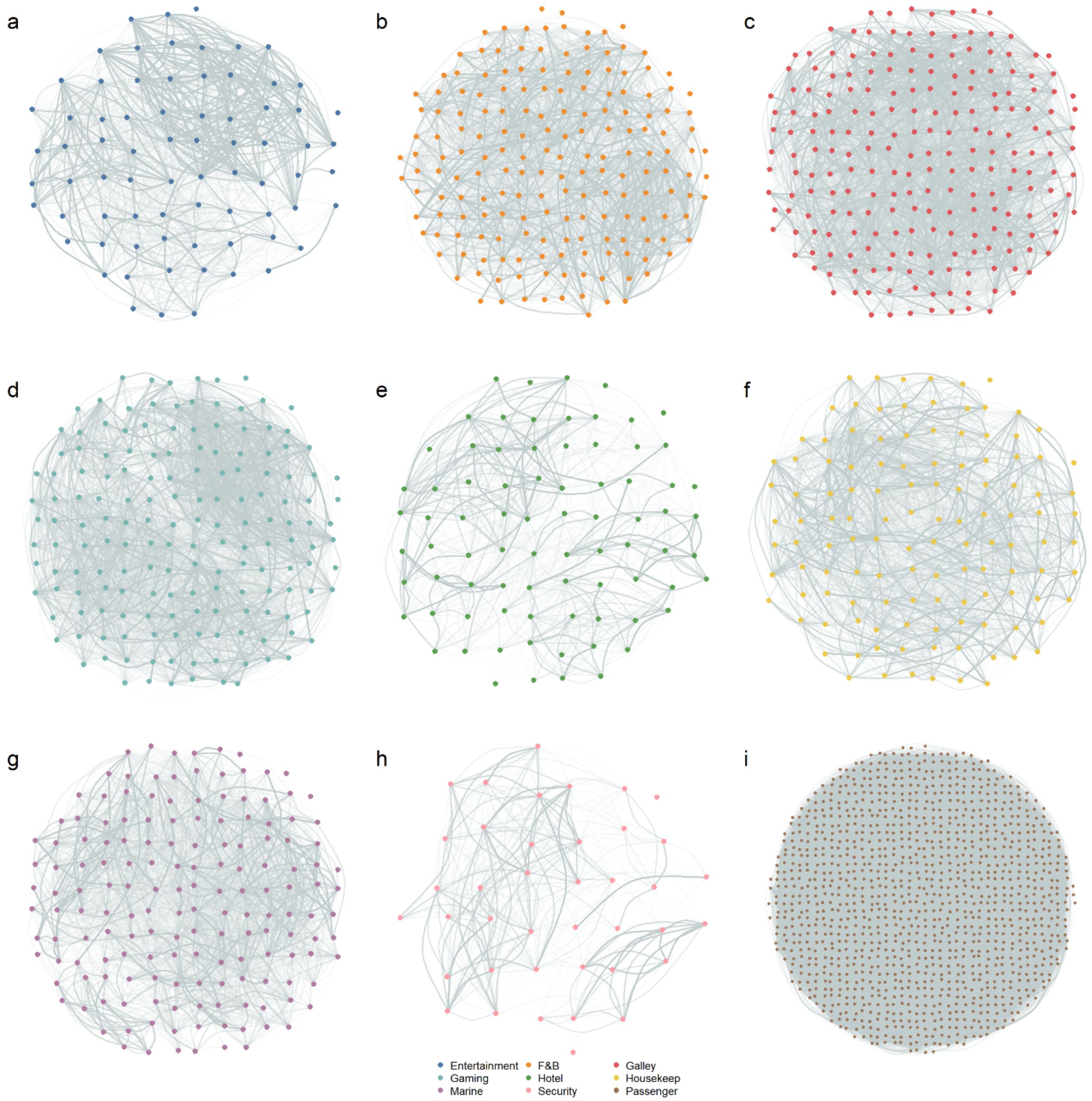

Supplementary Figure 2 Static intra-cohort contacts throughout the entire sailing, with crew from entertainment (a), F&B (b), galley (c), gaming (d), hotel services (e), housekeeping (f), marine (g), security and surveillance (h) departments and passengers (i). In addition, there were 77,107 unique pairs of crew contacts from different cohorts and 70,360 unique pairs of crew and passenger contacts but these links were not represented in this figure. Edge width and colour intensity of the edges correspond to the weights of a contact with the highest colour intensity as shown in the legend. Edge weights are a function of the proportion of days with recorded contact over a three-day sail period and the exponent transformation of the mean daily cumulative contact duration between two individuals.

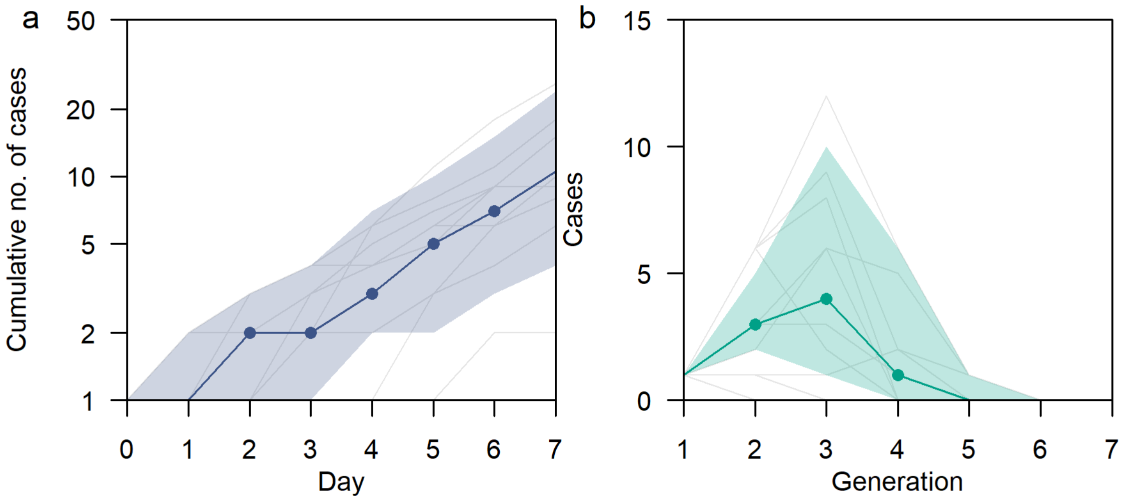

Supplementary Figure 3 (a) Cumulative cases by day of exposure and (b) number of cases in respective generations in the baseline scenario. Median (dots) and 95% intervals (shaded region) and outbreak trajectory for 10 selected simulations (grey lines) are shown.

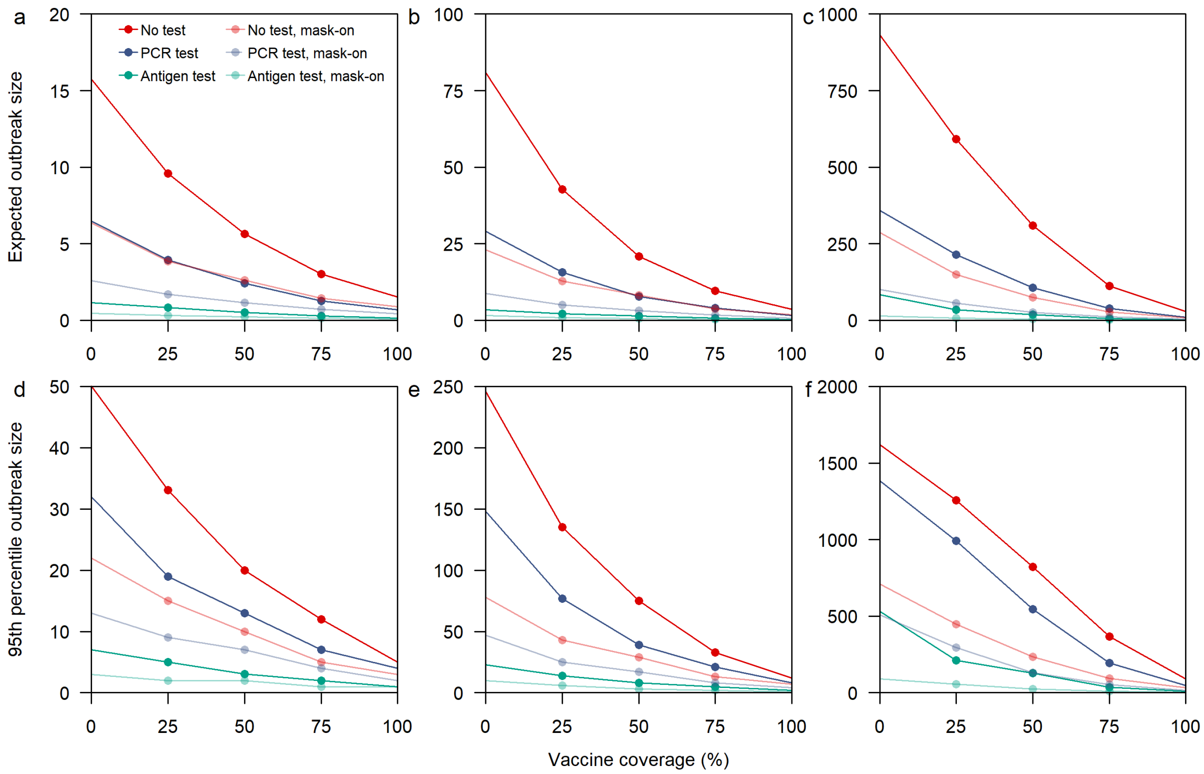

Supplementary Figure 4 Average and 95th percentile in outbreak size for varying interventions, vaccination coverage and assumption on network edge. Vaccines were assumed to confer 50% protection against infection and 50% lowered infectiousness for breakthrough infections in vaccinated individuals. Presymptomatic transmission was modelled to occur in 25% of the infections. (a, d) Edge weights vary based on the proportion of days with recorded interaction over a three-day sail period and duration of contact with weights increasing with days of interaction and contact time but reaches 95% saturation after 3 hours of contact, (b, e) same as (a, d) but reaches 95% saturation after 1 hour of contact, (c, f) edge weights vary based on proportion of days with recorded interaction.

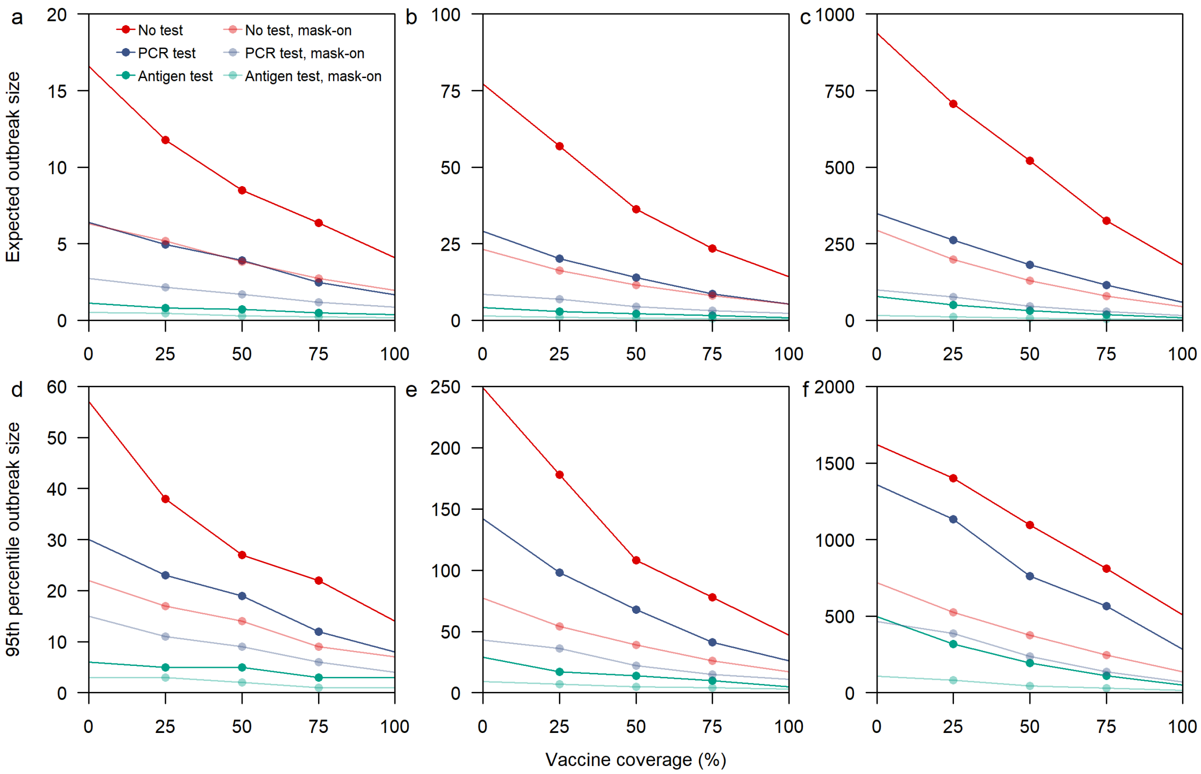

Supplementary Figure 5 Average and 95th percentile in outbreak size for varying interventions, vaccination coverage and assumption on network edge. Vaccine was assumed to confer 50% protection against infection but no lowered infectiousness. Presymptomatic transmission was modelled to occur in 25% of the infections. (a, d) Edge weights vary based on the proportion of days with recorded interaction over a three-day sail period and duration of contact with weights increasing with days of interaction and contact time but reaches 95% saturation after 3 hours of contact, (b, e) same as (a, d) but reaches 95% saturation after 1 hour of contact, (c, f) edge weights vary based on proportion of days with recorded interaction.

Relative to supplementary fig. 4, the expected outbreak size of all simulations increased across all vaccination coverage but the trend of outbreak size across varying coverage and differences between different combinations of interventions remains relatively unchanged

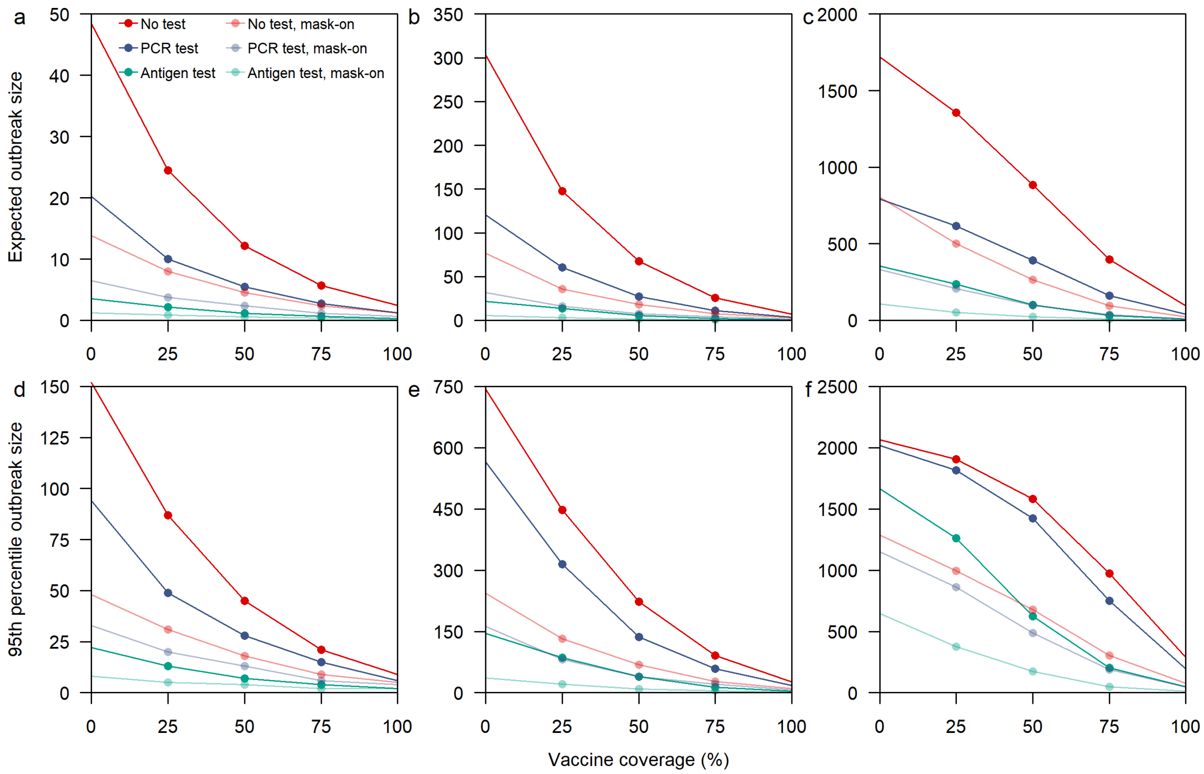

Supplementary Figure 6 Average and 95th percentile in outbreak size for varying interventions, vaccination coverage and assumption on network edge. Vaccine was assumed to confer 50% protection against infection and 50% lowered infectiousness. Presymptomatic transmission was modelled to occur in 50% of the infections. (a, d) Edge weights vary based on the proportion of days with recorded interaction over a three-day sail period and duration of contact with weights increasing with days of interaction and contact time but reaches 95% saturation after 3 hours of contact, (b, e) same as (a, d) but reaches 95% saturation after 1 hour of contact, (c, f) edge weights vary based on proportion of days with recorded interaction.

Relative to supplementary fig. 4, individuals with onset late into the event were able to generate more infections and drove up the expected outbreak sizes. Furthermore, the differences between a mask-off, once off PCR intervention and a mask-on baseline intervention widens with the former having lowered potential in identifying cases prior to the event. At low or no vaccine coverage, the 95th percentile outbreak size under mask-on interventions was lower than that for mask-off interventions with the latter approaching an outbreak size of 90%.

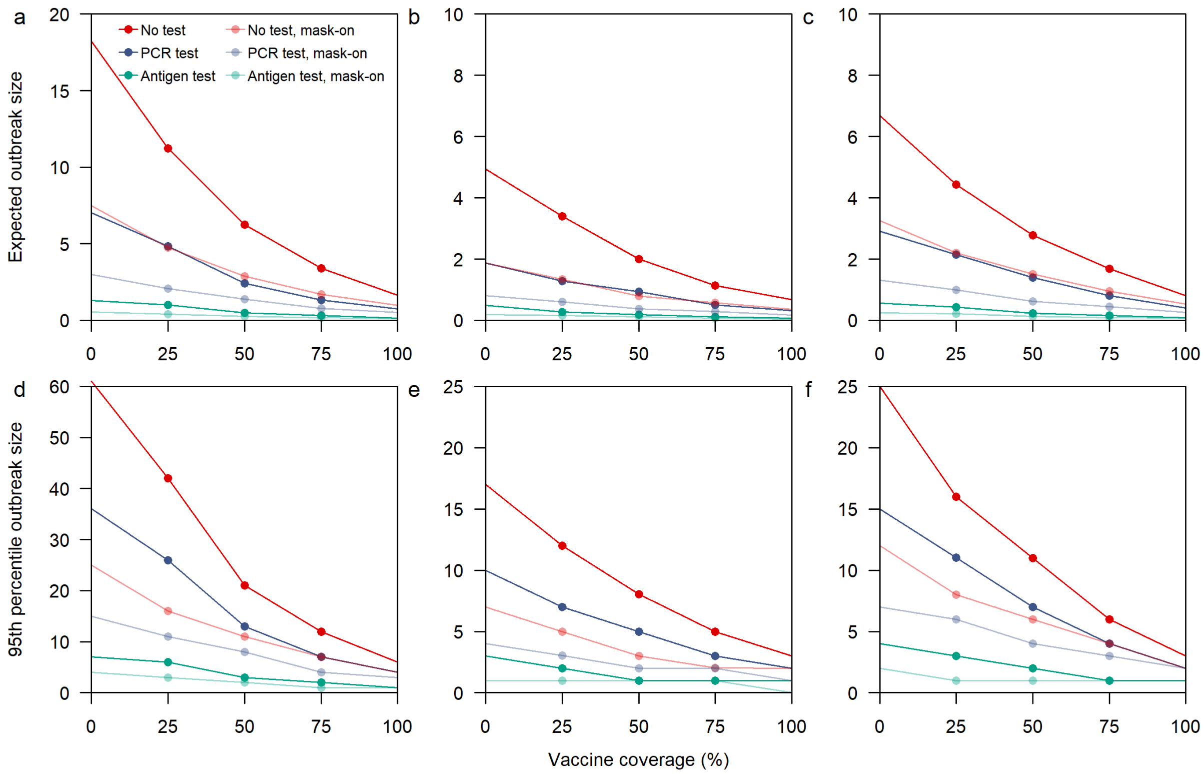

Supplementary Figure 7 Average and 95th percentile in outbreak size for varying interventions, vaccination coverage and for <u>different cruise sailings</u> on second (a, d), third (b, e) and fourth (c, f) sailing. General similarities in the results were found across all sailings (as potentially expected given their similarities in network structure). Vaccine was assumed to confer 50% protection against infection and 50% lowered infectiousness. Presymptomatic transmission was modelled to occur in 25% of the infections. Edge weights vary based on the proportion of days with recorded interaction over a three-day sail period and duration of contact with weights increasing with days of interaction and contact time but reaches 95% saturation after 3 hours of contact.

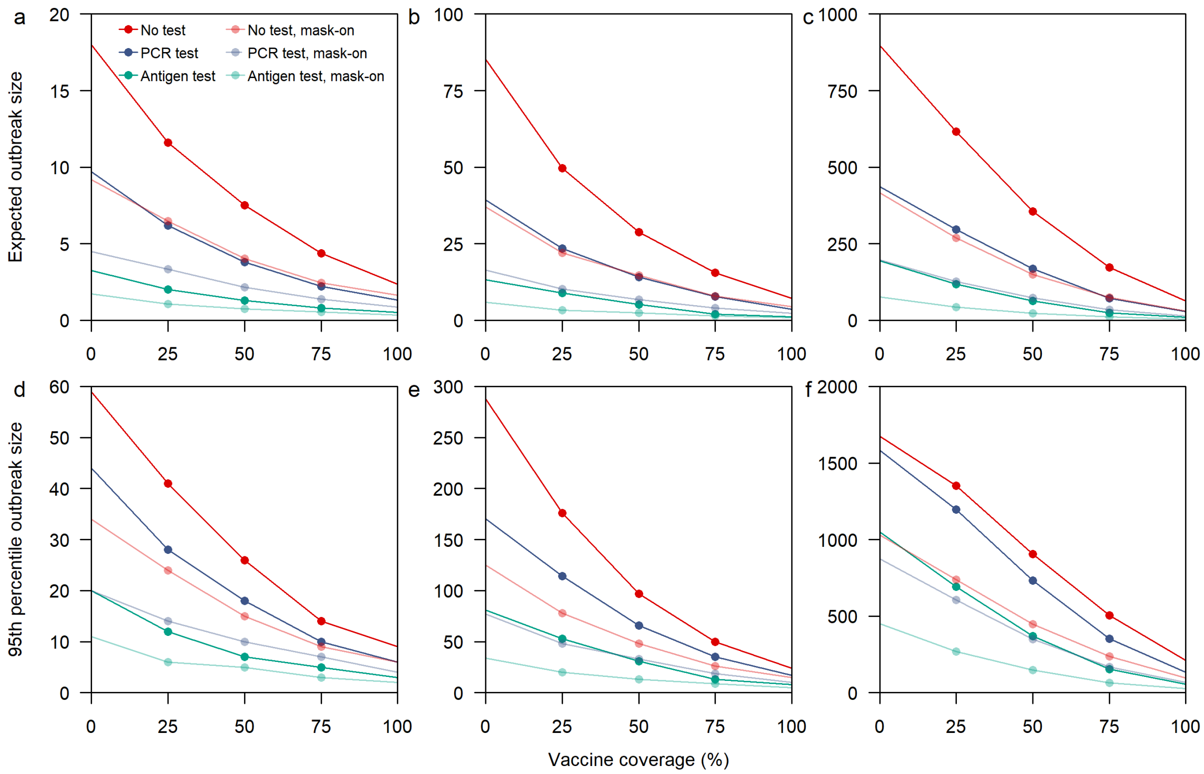

Supplementary Figure 8 Average and 95th percentile in outbreak size for varying interventions and vaccination coverage for outbreaks simulated based on a range of uncertainty in parameter values detailed in Table S1. (a, d) Daily edge weights vary based on the duration of contact with weights increasing with contact time but reaches 95% saturation after 3 hours of contact, (b, e) same as (a, d) but reaches 95% saturation after 1 hour of contact, (c, f) edge weights of 1 when interaction is recorded.

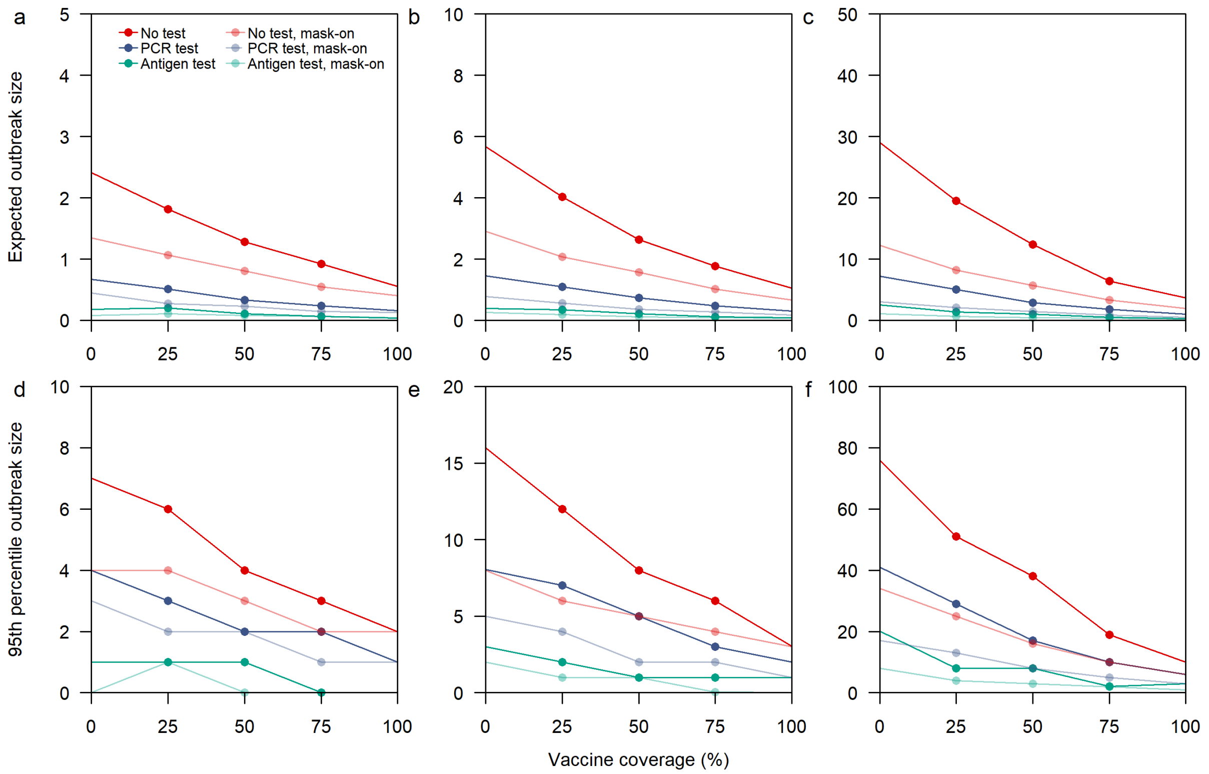

Supplementary Figure 9 Average and 95th percentile in outbreak size for varying interventions and vaccination coverage for outbreaks simulated on the temporal network for 3 days of sailing. Vaccines were assumed to confer 50% protection against infection and 50% lowered infectiousness for breakthrough infections in vaccinated individuals. Presymptomatic transmission was modelled to occur in 25% of the infections. (a, d) Daily edge weights vary based on the duration of contact with weights increasing with contact time but reaches 95% saturation after 3 hours of contact, (b, e) same as (a, d) but reaches 95% saturation after 1 hour of contact, (c, f) edge weights of 1 when interaction is recorded.

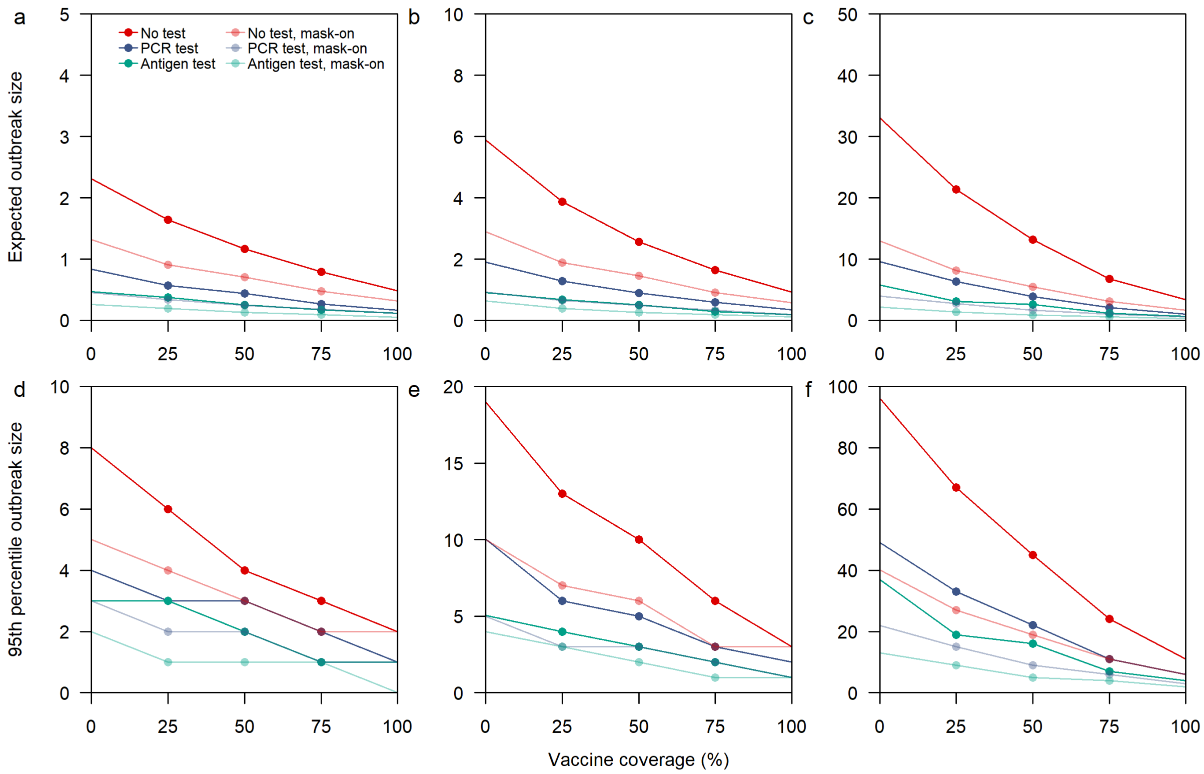

Supplementary Figure 10 Average and 95th percentile in outbreak size for varying interventions and vaccination coverage for outbreaks simulated on the static network for 3 days of sailing. Vaccines were assumed to confer 50% protection against infection and 50% lowered infectiousness for breakthrough infections in vaccinated individuals. Presymptomatic transmission was modelled to occur in 25% of the infections. (a, d) Daily edge weights vary based on the duration of contact with weights increasing with contact time but reaches 95% saturation after 3 hours of contact, (b, e) same as (a, d) but reaches 95% saturation after 1 hour of contact, (c, f) edge weights of 1 when interaction is recorded.

Supplementary Table 1.
Parameter uncertainty for Fig S8, assuming uniform distribution across the assumed values

Parameter	Assumed values	Details and references
Pre-symptomatic transmission	25-50%	[1]
Adherence to isolation when tested positive	60-100%	For scenarios involving testing only, we assume that there are available cabins for individuals to isolate given that cruises are operating at 50% capacity.<br><br>Lower bound based on self-reported adherence to isolation in the UK[2].
Relative risk of transmission by mask-off vaccinated, infected individual i	50-100%	Mean probability of transmitting infection reduces by 0-50%[3,4]**.**
Relative risk of acquiring infection by mask-off vaccinated, susceptible individual j	30-50%	Mean probability of acquiring infection reduces by 50-70%[4–9].
Relative risk of transmission when both infected individual i and susceptible individual j are wearing a mask	20-60%	Mean probability of infection reduces by about 40-80% when both the infected individual and susceptible contact are wearing a mask[10].

Supplementary Table 2.
Work functions of respective crew department

Department	Work functions
Entertainment	Cruise shows, live entertainment
Food & Beverage	From-end consumer facing food and beverages services
Galley	Back-end non-consumer facing galley, provision, stewarding
Gaming	Casinos, sports, arcade
Hotel	Hotel admin, front desk, embarkation training, spa, finance, IT, retail
Housekeep	Housekeeping, laundry
Marine	Deck, safety, security, medical, engineers, technicians, contractors
Security	Surveillance, security

Supplementary Note 1: CMMID COVID-19 Working Group funding

The following funding sources are acknowledged as providing funding for the working group authors. This research was partly funded by the Bill & Melinda Gates Foundation (INV-001754: MQ; INV-003174: KP, MJ, YL; INV-016832: SRP; NTD Modelling Consortium OPP1184344: CABP, GFM; OPP1139859: BJQ; OPP1191821: KO'R). BMGF (INV-016832; OPP1157270: KA). CADDE MR/S0195/1 & FAPESP 18/14389-0 (PM). EDCTP2 (RIA2020EF-2983-CSIGN: HPG). ERC Starting Grant (#757699: MQ). ERC (SG 757688: CJVA, KEA). This project has received funding from the European Union's Horizon 2020 research and innovation programme - project EpiPose (101003688: AG, KLM, KP, MJ, RCB, WJE, YL). FCDO/Wellcome Trust (Epidemic Preparedness Coronavirus research programme 221303/Z/20/Z: CABP). This research was partly funded by the Global Challenges Research Fund (GCRF) project 'RECAP' managed through RCUK and ESRC (ES/P010873/1: CIJ). HDR UK (MR/S003975/1: RME). HPRU (This research was partly funded by the National Institute for Health Research (NIHR) using UK aid from the UK Government to support global health research. The views expressed in this publication are those of the author(s) and not necessarily those of the NIHR or the UK Department of Health and Social Care200908: NIB). MRC (MR/N013638/1: EF; MR/V027956/1: WW). Nakajima Foundation (AE). NIHR (16/136/46: BJQ; 16/137/109: BJQ, FYS, MJ, YL; 1R01AI141534-01A1: DH; NIHR200908: LACC, RME; NIHR200929: CVM, FGS, MJ, NGD; PR-OD-1017-20002: AR, WJE). Royal Society (Dorothy Hodgkin Fellowship: RL). UK DHSC/UK Aid/NIHR (PR-OD-1017-20001: HPG). UK MRC (MC_PC_19065 - Covid 19: Understanding the dynamics and drivers of the COVID-19 epidemic using real-time outbreak analytics: NGD, RME, SC, WJE, YL; MR/P014658/1: GMK). UKRI (MR/V028456/1: YJ). Wellcome Trust (206250/Z/17/Z: TWR; 206471/Z/17/Z: OJB; 208812/Z/17/Z: SC, SFlasche; 210758/Z/18/Z: JDM, JH, KS, SA, SFunk, SRM; 221303/Z/20/Z: MK). No funding (DCT, SH).

Supplementary References

1. Buitrago-Garcia, D. *et al.* Occurrence and transmission potential of asymptomatic and presymptomatic SARS-CoV-2 infections: A living systematic review and meta-analysis. *PLoS Med* **17**, e1003346 (2020).
2. Office for National Statistics. Coronavirus (COVID-19) latest insights - Office for National Statistics. https://www.ons.gov.uk/peoplepopulationandcommunity/healthandsocialcare/conditionsanddiseases/articles/coronaviruscovid19latestinsights/lifestyle (2021).
3. Harris, R. J. *et al.* Effect of Vaccination on Household Transmission of SARS-CoV-2 in England. *New England Journal of Medicine* **385**, 759–760 (2021).
4. Singanayagam, A. *et al.* Community transmission and viral load kinetics of the SARS-CoV-2 delta (B.1.617.2) variant in vaccinated and unvaccinated individuals in the UK: a prospective, longitudinal, cohort study. *The Lancet Infectious Diseases* **0**, (2021).
5. Elliott, P. *et al. REACT-1 round 13 final report: exponential growth, high prevalence of SARS-CoV-2 and vaccine effectiveness associated with Delta variant in England during May to July 2021*. http://spiral.imperial.ac.uk/handle/10044/1/90800 (2021).
6. Nanduri, S. Effectiveness of Pfizer-BioNTech and Moderna Vaccines in Preventing SARS-CoV-2 Infection Among Nursing Home Residents Before and During Widespread Circulation of the SARS-CoV-2 B.1.617.2 (Delta) Variant — National Healthcare Safety Network, March 1–August 1, 2021. *MMWR Morb Mortal Wkly Rep* **70**, 1163–1166 (2021).
7. Fowlkes, A. Effectiveness of COVID-19 Vaccines in Preventing SARS-CoV-2 Infection Among Frontline Workers Before and During B.1.617.2 (Delta) Variant Predominance — Eight U.S. Locations, December 2020–August 2021. *MMWR Morb Mortal Wkly Rep* **70**, 1167–1169 (2021).
8. Bernal, J. L. *et al.* Effectiveness of COVID-19 vaccines against the B.1.617.2 variant. *medRxiv* 2021.05.22.21257658 (2021) doi:10.1101/2021.05.22.21257658.
9. Polack, F. P. *et al.* Safety and Efficacy of the BNT162b2 mRNA Covid-19 Vaccine. *New England Journal of Medicine* **383**, 2603–2615 (2020).
10. Chu, D. K. *et al.* Physical distancing, face masks, and eye protection to prevent person-to-person transmission of SARS-CoV-2 and COVID-19: a systematic review and meta-analysis. *The Lancet* **395**, 1973–1987 (2020).
