# Supplementary material for: Using high-resolution contact networks to evaluate SARS-CoV-2 transmission and control in large-scale multi-day events
Source: Nat Commun. 2022 Apr 12;13:1956. doi: 10.1038/s41467-022-29522-y (PMC9005731; doi:10.1038/s41467-022-29522-y)
Supplement: Supplementary file 3 — Reporting Summary [file 41467_2022_29522_MOESM3_ESM.pdf]

nature portfolio

Corresponding author(s): Rachael Pung

Last updated by author(s): Feb 8, 2022

Reporting Summary

Nature Portfolio wishes to improve the reproducibility of the work that we publish. This form provides structure for consistency and transparency in reporting. For further information on Nature Portfolio policies, see our Editorial Policies and the Editorial Policy Checklist.

Statistics

For all statistical analyses, confirm that the following items are present in the figure legend, table legend, main text, or Methods section.

n/a	Confirmed	
☐	☒	The exact sample size (n) for each experimental group/condition, given as a discrete number and unit of measurement
☐	☒	A statement on whether measurements were taken from distinct samples or whether the same sample was measured repeatedly
☐	☒	The statistical test(s) used AND whether they are one- or two-sided<br>*Only common tests should be described solely by name; describe more complex techniques in the Methods section.*
☒	☐	A description of all covariates tested
☒	☐	A description of any assumptions or corrections, such as tests of normality and adjustment for multiple comparisons
☐	☒	A full description of the statistical parameters including central tendency (e.g. means) or other basic estimates (e.g. regression coefficient) AND variation (e.g. standard deviation) or associated estimates of uncertainty (e.g. confidence intervals)
☐	☒	For null hypothesis testing, the test statistic (e.g. F, t, r) with confidence intervals, effect sizes, degrees of freedom and P value noted<br>*Give P values as exact values whenever suitable.*
☒	☐	For Bayesian analysis, information on the choice of priors and Markov chain Monte Carlo settings
☒	☐	For hierarchical and complex designs, identification of the appropriate level for tests and full reporting of outcomes
☒	☐	Estimates of effect sizes (e.g. Cohen's d, Pearson's r), indicating how they were calculated

Our web collection on statistics for biologists contains articles on many of the points above.

Software and code

Policy information about availability of computer code

Data collection	Publicly available code provided in the analysis repository: https://doi.org/10.5281/zenodo.6009027
Data analysis	Publicly available code provided in the analysis repository: https://doi.org/10.5281/zenodo.6009027

For manuscripts utilizing custom algorithms or software that are central to the research but not yet described in published literature, software must be made available to editors and reviewers. We strongly encourage code deposition in a community repository (e.g. GitHub). See the Nature Portfolio guidelines for submitting code & software for further information.

Data

Policy information about availability of data

All manuscripts must include a data availability statement. This statement should provide the following information, where applicable:

- Accession codes, unique identifiers, or web links for publicly available datasets
- A description of any restrictions on data availability
- For clinical datasets or third party data, please ensure that the statement adheres to our policy

Publicly available data provided in the analysis repository: https://doi.org/10.5281/zenodo.6009027

Field-specific reporting

Please select the one below that is the best fit for your research. If you are not sure, read the appropriate sections before making your selection.

☐ Life sciences ☒ Behavioural & social sciences ☐ Ecological, evolutionary & environmental sciences

For a reference copy of the document with all sections, see nature.com/documents/nr-reporting-summary-flat.pdf

Behavioural & social sciences study design

All studies must disclose on these points even when the disclosure is negative.

Study description	Quantitative contact network data collection
Research sample	Study involves passengers and crews onboard cruises in Singapore. There is an approximately equal representation of male and females among passengers. Passengers age from less than 12 years old to more than 70 years old with an interquartile range of 35 to 63 years old. Participants are representative of cruise travelers during the COVID-19 pandemic. Given the large amount of granular contact data to collect and process, the study focuses on four cruise sailings spread out over 4 months.
Sampling strategy	Data from all passengers and crew onboard a sailing was used. No sampling was performed.
Data collection	All participants were carrying a bluetooth contact tracing device. The researcher is not present during each of the sails to avoid any influence to the data collection process.
Timing	Nov 2020 to Feb 2021
Data exclusions	No data were excluded from the analyses
Non-participation	No participants dropped out.
Randomization	The impact of outbreak interventions were studied in silico and the initial infected individuals, disease characteristics of all infected persons, and adherence to interventions were randomised within each simulation.

Reporting for specific materials, systems and methods

We require information from authors about some types of materials, experimental systems and methods used in many studies. Here, indicate whether each material, system or method listed is relevant to your study. If you are not sure if a list item applies to your research, read the appropriate section before selecting a response.

Materials & experimental systems

n/a	Involved in the study	
☒	☐	Antibodies
☒	☐	Eukaryotic cell lines
☒	☐	Palaeontology and archaeology
☒	☐	Animals and other organisms
☒	☐	Human research participants
☒	☐	Clinical data
☒	☐	Dual use research of concern

Methods

n/a	Involved in the study	
☒	☐	ChIP-seq
☒	☐	Flow cytometry
☒	☐	MRI-based neuroimaging
